# Immunologic changes underlying to an egg OIT protocol in a pediatric population

**DOI:** 10.1186/2045-7022-5-S3-O15

**Published:** 2015-03-30

**Authors:** Laura Perezábad, Marta Reche, Teresa Valbuena, Antonia Padial, Cristina Pascual, Rosina López-Fandiño, Elena Molina, Iván López-Expósito

**Affiliations:** 1 Department of Bioactivity and Food Analysis, Institute for Food Science Research (CIAL) (CSIC-UAM), Madrid, Spain; 2 Allergy Service, Infanta Sofia Hospital, San Sebastian de los Reyes, Madrid, Spain

## Background

Egg allergy is one of the most common food allergies in childhood with an estimated prevalence of 2.6 %. Oral immunotherapy (OIT) appears as a promising approach to induce egg-tolerance. However, the immune mechanisms underlying this treatment are not completely known. The purpose of the study was to compare the immune responses against egg allergens of a pediatric population under an OIT protocol before and after the protocol was finished.

## Methods

A group of 20 infants with egg allergy confirmed by egg-specific IgE levels and positive DBPCT were subjected to an egg OIT protocol for an average time of 11.5 months. Mean age was 10.5 years old. PBMCs were isolated from peripheral blood and stimulated during 7 days with 200 μg/mL of ovalbumin (OVA). Culture supernatants were analyzed for IL-5, IL-13, IFN-γ, TNF-α and IL-10 production by Cytometric Bead Array (CBA). RNA was isolated from PBMC´s and qPCR was carried out to quantify transcription factors FoxP3, GATA3 and Tbet.

## Results

12 patients completed successfully the OIT protocol being able to tolerate 32 mL of egg white (EW). OVA, ovomucoid (OM) and EW-specific IgE levels were significantly lower after the OIT protocol (P<0.1 for OM and OVA and P<0.01 for EW). Moreover, OVA, OM and EW-specific IgG4 were significantly higher once the OIT was completed (P<0.07).

CBA results showed an increased production of OVA-specific IL10. In addition, a trend towards a lower OVA-specific IL-5 and IL-13 production and higher IFN-1 and TNF-1 levels was found. Regarding qPCR analysis, transcription factors FoxP3 and Tbet were upregulated in 55.5% and 77.7% of the patients respectively.

## Conclusion

60% of the patients were able to tolerate a whole egg after the OIT protocol used in this study. A significant shift in the humoral response towards impaired OVA, OM and EW specific-IgE production and increased IgG4 levels was observed. This was accompanied by a significant enhancement in OVA-specific IL10 production by T cells together with an upregulation in the transcription factors FoxP3 and Tbet. The relevant role for the Treg population and for the balance Th1 to Th2 cells in tolerance development is underscored.

